# Perceived vulnerability, fear of covid-19, and psychological distress of military hospital healthcare workers

**DOI:** 10.1192/j.eurpsy.2024.553

**Published:** 2024-08-27

**Authors:** C. Papathanasiou, D. Christodoulias

**Affiliations:** ^1^Department of Psychology, Panteion University of Social and Political Sciences, Athens; ^2^School of Social Sciences, Hellenic Open University, Patras, Greece

## Abstract

**Introduction:**

The healthcare workers of military hospitals are actively involved in the fight against covid-19, as part of the national healthcare systems. Therefore, these health professionals may experience symptoms of psychological distress.

**Objectives:**

The study of sociodemographic characteristics and pandemic-related psychosocial factors that affect the psychological distress of healthcare professionals in a military hospital.

**Methods:**

134 health professionals participated (- 34.3% doctors, 53% nurses and 12.7% other staff ). A cross-sectional study was conducted using the DASS-21, PVDS, and FCV-19S questionnaires. Demographic variables were also collected. The data was analyzed using student’s t-test and Mann-Whitney test, analysis of variance and Kruskal-Wallis test, Pearson’s correlation coefficient and Spearman’s correlation coefficient, as well as multivariate linear regression.

**Results:**

21.64%, 17.91%, and 16.42% of the sample showed symptoms of depression, anxiety, and stress respectively. A significant correlation emerged between all three dimensions with perceived infectibility and fear of covid-19. Contact with a possible covid-19 patient, female gender, marriage, underlying diseases, increased working hours were found as stressors. The mean values of perceived infectibility and germs aversion were 3.4 and 4.9, respectively. A significant correlation was found between the two subscales with fear of covid-19 (p=0.001 and <0.001 respectively). Participants who had undergone psychotherapy in the past had a higher score of perceived infectibility (p=0.024). Women and staff in the pathological sector showed greater aversion to germs (p=0.040 and 0.001 respectively). Educational level and working hours were negatively correlated with germs aversion (p=0.037 and 0.044 respectively). The mean of fear of covid-19 was 14.5, with 14.2% of the population being above the scale average. Fear of covid-19 showed a positive correlation with female gender, age, family, contact with a possible positive case. It was negatively correlated with the medical staff, the educational level, and the employees in a covid-19 clinic. According to the results of the multivariate linear regression analyses: (i) The increase in educational level was associated with a decrease in the fear for covid-19 score (p=0.026); (ii) The increase in perceived infectibility score was associated with an increase in the fear for covid-19 score (p<0.001); (iii) The increase in germs aversion score was associated with an increase in fear for covid-19 score (p=0.014).

**Conclusions:**

The findings confirm the presence of psychological distress on the healthcare workers of the hospital and its dependence on perceived infectibility and fear of covid-19.

**Disclosure of Interest:**

None Declared

